# A Permanent Magnet Ferromagnetic Wear Debris Sensor Based on Axisymmetric High-Gradient Magnetic Field

**DOI:** 10.3390/s22218282

**Published:** 2022-10-28

**Authors:** Bin Fan, Yong Liu, Peng Zhang, Lianfu Wang, Chao Zhang, Jianguo Wang

**Affiliations:** 1 College of Mechanical & Electrical Engineering, Inner Mongolia Agricultural University, Hohhot 010018, China; 2 State Key Laboratory of Smart Manufacturing for Special Vehicles and Transmission System, Baotou 014030, China; 3 Inner Mongolia Key Laboratory of Intelligent Diagnosis and Control of Mechatronic Systems, Inner Mongolia University of Science & Technology, Baotou 014010, China

**Keywords:** permanent magnet, fault diagnosis, finite element, wear debris sensor

## Abstract

The detection of wear debris in lubricating oil is effective for determining current equipment operating conditions for fault diagnosis. In this paper, a permanent magnet ferromagnetic wear debris sensor is proposed that is composed of a compact structure and a detection coil that generates an induced voltage when wear debris passes through a magnetic field. A three-dimensional model of the sensor is established, the internal axisymmetric high-gradient magnetic field of the sensor is analyzed, and a mathematical model of the sensor signal is proposed. The effects of the air gap structure of the sensor and the relative permeability, velocity, and volume of the wear debris on the sensor performance are analyzed. The correctness of the theoretical results is proven by single particle experiments, and the sensor is calibrated to achieve quantitative analysis of the wear debris.

## 1. Introduction

With the continuous improvement of the degree of information available for large-scale machinery and equipment in the industrial field, prognostics and health management (PHM) technology has received increasing attention [1]. Fault diagnosis technology is the basis of PHM, which can be used to effectively monitor the operational status of mechanical equipment, among which vibration monitoring [2] and oil monitoring [3] have been widely used. Wear is one of the factors that induces mechanical equipment faults [4], and vibration monitoring methods can be used to realize better detection when mechanical equipment is subject to serious wear; however, in such cases the vibration signal is transmitted through multiple interfaces, its energy attenuation is large, and the energy loss and poor working environment can seriously affect the vibration monitoring effect [5]. Oil monitoring technology relies on monitoring the wear debris generated by the friction of mechanical parts in the lube oil circuit. The size, material, and number of wear debris can be used to directly characterize the wear status of equipment [6]. Various types of wear debris sensors have been developed according to different detection principles [7,8,9,10,11,12]. Among them, inductive wear debris sensors with the advantages of simple structure, quick response, and strong anti-interference properties, have occupied an important position in the field of online wear debris monitoring [13,14,15].

Inductive wear debris sensors are mainly divided into alternating magnetic field types and static magnetic field types. The principle of operation for an alternating magnetic field sensor is based on the dual effects of eddy current and magnetization of wear debris that change the distribution of the magnetic field to achieve the monitoring of wear debris. An alternating magnetic field induction type sensor can be used to simultaneously consider the detection of ferromagnetic and nonferromagnetic wear debris. Its simple probe structure is suitable for aviation and other application fields that require a light-weight design [16]. Gastops [17] proposed the classic three solenoid coil structure sensor, which greatly improves the detection accuracy of wear particles on the basis of a single-coil or double-coil sensor. This structure has been widely recognized by researchers and has been applied in the field of aviation. Zeng [18] et al. proposed a highly sensitive multiparameter microsensor consisting of a microfluidic chip and a sensing unit, which can be used to detect 33 μm iron particles and 90 μm copper particles with an inductive detection unit and 100 μm water droplets and 180 μm air bubbles in hydraulic oil with a capacitive detection unit. Du et al. [19] proposed a microfluidic wear debris sensor based on a planar coil, with which 50 µm diameter iron debris and 100 µm diameter copper de-bris can be detected in a 250 μm high and 500 μm wide pipe. The addition of a capacitor to the induction coil of the inductive sensor to form a parallel LC resonant circuit with a unique resonant frequency enabled the detection of 20 μm iron particles and 55 μm copper particles [20]. Zhu [21] et al. proposed a 3 × 3 planar coil sensing array by connecting an LCR measurement circuit in parallel to each induction coil. Using a simultaneous sampling method, 50 μm ferromagnetic wear debris and 150 μm nonferromagnetic wear de-bris can be detected. Ren et al. [22] proposed a sensor with two or more induction coils and only one excitation coil, which can detect 120 μm iron particles and 210 μm copper particles. A particle sensor based on a harmonic magnetic field is a classic type and is becoming more and more developed. However, its inherent flaws are inevitable: (1) it is not possible to improve ferro and non-ferromagnetic particles’ detection accuracy at the same time by changing excitation frequency [23], as the opposite effects of eddy currents and magnetization in ferromagnetic particles; (2) signal extraction is relatively hard, and the common method (demodulation circuits and impedance analyzers) is not conducive to practical industrial applications [24,25].

In a static magnetic field, a ferromagnetic particle is only influenced by the magnetization effect. Hence, this type of sensor has a higher detection accuracy for ferro-particles. As there is no more signal process but amplification, the signal extraction can be more convenient and cheaper. Moreover, the sensor’s application is also wide, for the most tribo-pairs are ferro-products. Feng et al. [26] proposed a sensor with an axial high-gradient static magnetic field, which can detect 25 μm iron particles. To further improve the detection accuracy, they [27] later proposed a sensor based on a rotationally symmetric high-gradient magnetostatic field, which consists of a magnetic ring with two cylindrical poles and a magnetic housing. This sensor can detect 13 μm iron particles in a 10 mm pipe. Hong et al. [28] proposed an inductive method based on a radial magnetic field to improve the uniformity of the magnetic field, which can achieve the detection of 20 μm iron particles in a 20 mm pipe. Compared with the sensor with an alternating magnetic field, the static magnetic field sensor has a higher detection accuracy for ferro-particles and a simpler signal processing circuit. However, the conventional static magnetic field inductive sensor usually contains bulky iron core components, which make it difficult to realize a lightweight design for application in the aerospace and other fields, and the quality of the magnetic field is greatly affected by the noise of the excitation power supply. Hong et al. [29] developed a permanent magnet structure-based wear debris sensor in which the magnetic field generated by a permanent magnet is more stable than that generated by an electromagnetic coil. However, the magnetic field strength of the sensor is relatively low, and the magnetic circuit is relatively long, which is not conducive to improving the detection of small wear debris. Zheng Y [30] et al. proposed a passive sensor based on a permanent magnetic ring, whose structural design greatly reduces the volume and weight of the sensor. However, this sensor has too much magnetic leakage, resulting in very little magnetic field in the sensing area, which requires higher performance of the magnetic ring.

To solve the problem of magnetic field instability and excessive volume of inductive sensors, this paper proposes a permanent magnet axisymmetric high-gradient magnetic field inductive wear debris sensor with a compact sensor structure, no external power supply, no energy loss, and a stable magnetic field generated by permanent magnets. The main contributions are as follows:

- The chamfered structure of the sensor can enhance the strength and axial gradient of the magnetic field in the flow channel, which improves the sensing performance of the wear debris sensor;
- A mathematical model and a simulation model of the sensor is established. The influence of related design parameters on sensor performance was analyzed and the simulation was verified by single wear debris experiments;
- Lube oil cycle experiments were carried out, it was found that the sensor can effectively detect iron particles of 50 μm in size in lube oil at a flow rate of 500 mL/min.

## 2. Structure, Principle and Model

### 2.1. Sensor Structure

**Figure 1:**
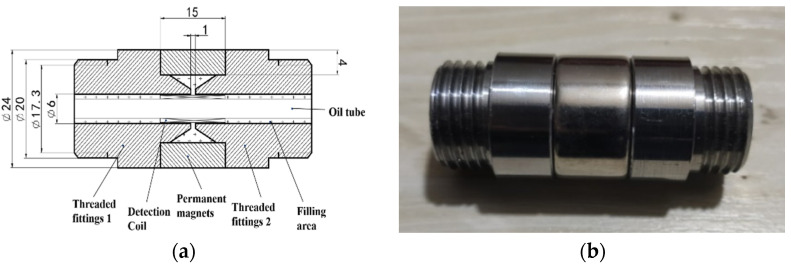
Structure of sensor: (**a**) structure, (**b**) sensor.

The structure of the sensor proposed in this paper is shown in Figure 1. It consists of a ring-shaped permanent magnet, two threaded joints, an induction coil, a filling area, and an oil tube. One end of the connector has a national standard external thread for easy installation. The two threaded joints are set on both sides of the ring-shaped permanent magnets, and there is an air gap between the joints, which generates a high-gradient magnetic field. The permanent magnet is composed of a NdFeB material of type N38.

### 2.2. Magnetic Field Analysis

**Figure 2:**
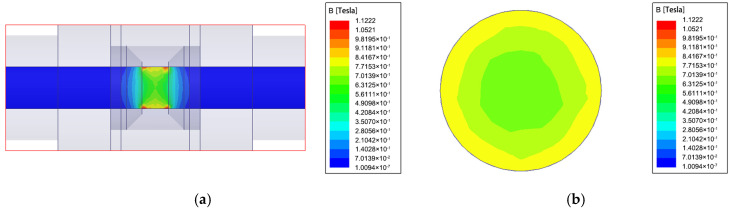
Static axisymmetric high-gradient static magnetic field analysis: (**a**) magnetic flux density in the axial direction and (**b**) magnetic flux density in the radial direction.

The static magnetic field of the three-dimensional model of the sensor was analyzed in Maxwell to investigate the distribution of the magnetic field inside the sensor. Figure 2a shows the distribution of the magnetic flux density in the axial middle section of the oil tube, and Figure 2b shows the distribution of the magnetic flux density in the radial direction. It can be found that in the axial direction of the sensor channel, there is magnetic flux leakage only in the local air gap, the magnetic field gradient is obvious, and the magnetic field has an axisymmetric distribution. In the radial direction of the sensor channel, the magnetic field distribution is obvious, and it also shows axisymmetric distribution.

**Figure 3:**
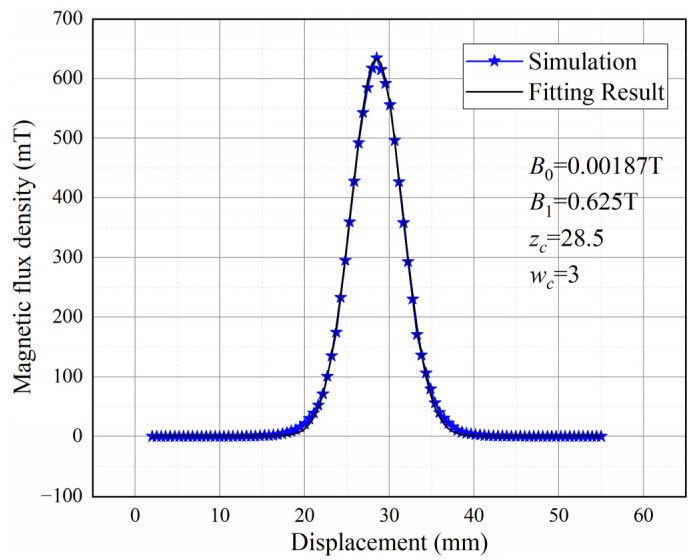
Magnetic flux density in the axial direction.

In this paper, it is considered that the wear debris passes along the axis of the flow channel. The magnetic field on the axis is nonlinearly fitted by the GaussAmp function. Figure 3 shows the curve for the magnetic flux density in the axial direction.

(1) $$B_{p}=\left\{B_{0}+B_{1}\exp\left[-0.5{\left(\frac{z-z_{c}}{w_{c}}\right)}^{2}\right]\right\}$$

### 2.3. Detetion Principle and Inductive Voltage Model

The NdFeB material has the advantages of high remanence, high coercivity and high magnetic performance, which forms a stable magnetic field inside the sensor oil tube. The sensor proposed in this paper is used for the monitoring of metal wear debris in lubricating oil. When metal wear debris passes through the induction coil of the sensor, it results in a change in the internal magnetic flux, and according to Faraday’s law of electromagnetic induction, the induced voltage generated by the coil can be expressed as:

(2) $$u=N\underset{\Delta t\to 0}{lim}\frac{\Delta \Phi }{\Delta t}$$

In a static magnetic field, the ferro-particle can be magnetized and generates a magnetized field outside the particle. The magnetized field owns the same direction with the external static field. When the particle passes through the induction region, the field in the induction region is the sum of the magnetized field and the external static field. Which changes the magnetic flux of the inductive coil, resulting in an induced voltage.

The particle’s magnetized field can be obtained by the metal particle’s magnetic vector potential. Take the center of a spherical metal particle as the origin of coordinates, the direction of the external magnetic field as the *z* axis, the magnetic vector potential $A_{\varphi }$ of a spherical metal particle magnetized by a static magnetic field is as follows [31]:

(3) $$A_{\varphi }=\frac{\mu_{r}-1}{\mu_{r}+2}\cdot \frac{a^{3}}{r^{2}}B_{0}\sin\theta$$

where $\mu_{r}$ is the relative permeability of the particle, a is the radius of the particle, r is r-axis component of arbitrary position in sphere coordinates, and $B_{0}$ is the external static magnetic field intensity.

The magnetic induction intensity of the debris scattering field can be obtained from the curl of the magnetic vector potential:

(4) $$\vec{B}=\nabla \times \vec{A}=\frac{1}{r^{2}\sin\theta }\left|\begin{array}{ccc} {\vec{e}}_{r} & r{\vec{e}}_{\theta } & r\sin\theta {\vec{e}}_{\varphi }\\ \frac{\partial }{\partial r} & \frac{\partial }{\partial \theta } & \frac{\partial }{\partial \varphi }\\ 0 & 0 & r\sin\theta \cdot A_{\varphi } \end{array}\right|$$

the component in the *z* axis direction is:

(5) $$B_{z}^{\prime }=\frac{\left(\mu_{r}-1\right)a^{3}}{\left(\mu_{r}+2\right)r^{3}}B_{p}\left(3\cos^{2}\theta -1\right)$$

where $B_{p}$ is the magnetic field intensity on the axis of the permanent magnet ring.

**Figure 4:**
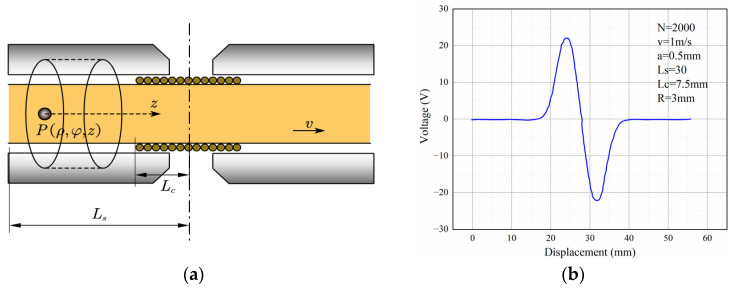
Mathematical model: (**a**) coordinate system when debris moving in the sensor; (**b**) induced voltage.

To obtain the induced voltage of the coil, we established a cylindrical coordinate system, as shown in Figure 4a. The magnetic flux in the induction region is obtained by integrating the magnetic induction intensity in the coil:

(6) $$\Phi =\iint B_{z}^{\prime }dSdz=\frac{\pi \left(\mu_{r}-1\right)Na^{3}}{\left(\mu_{r}+2\right)L_{c}}\cdot B_{p}\left(\frac{z_{2}}{\sqrt{R^{2}+z_{2}^{2}}}-\frac{z_{1}}{\sqrt{R^{2}+z_{1}^{2}}}\right)$$

where $z_{1}=L_{s}-L_{c}-vt$, $z_{2}=z_{1}+2L_{c}$, $R_{c}$ is the coil radius, and N  is the number of coils turns. $L_{s}$ is the half length of the debris movement path, $L_{c}$ is the half length of the coil, v is the velocity of the debris.

Then, substitute (6) into (1), the induced voltage is expressed as (7). Figure 4b shows the mathematical model curve, and the parameters are set as shown. From the mathematical model, it can be found that the voltage is proportional to the volume of the wear debris ($a^{3}$) and the magnetic flux density.

(7) $$u=\frac{\pi Na^{3}vB_{p}}{L_{c}}\left[\frac{vt-z_{c}}{w_{c}^{2}}\left(\frac{z_{2}}{\sqrt{R^{2}+z_{2}^{2}}}-\frac{z_{1}}{\sqrt{R^{2}+z_{1}^{2}}}\right)+\left(\frac{R^{2}}{\sqrt{{\left(R^{2}+z_{2}^{2}\right)}^{3}}}-\frac{R^{2}}{\sqrt{{\left(R^{2}+z_{1}^{2}\right)}^{3}}}\right)\right]$$

## 3. Simulation and Analysis of Factors Affecting Sensing Performance

To guide the sensor design, we discussed the effect of relevant parameters on the sensing performance through finite element simulations. The parameters discussed in the simulations are shown in the following Table 1.

### 3.1. Effect of Air Gap Distance

**Figure 5:**
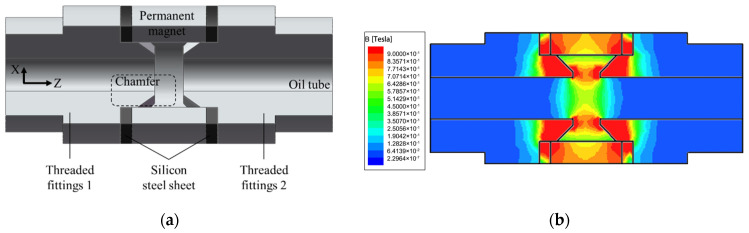
Three-dimensional simulation in ANSYS Maxwell: (**a**) structure; (**b**) cross section of simulation result.

To optimize the high-gradient magnetic field inside the sensor, the effect of the distance of the air gap and the size of the chamfer of the threaded joint on the magnetic field intensity at the axial position of the sensor was analyzed. The length of the air gap was adjusted by a pair of silicon steel sheet. The simulation structure in ANSYS Maxwell is shown as Figure 5a, and the cross section of the 3D simulation results is shown in Figure 5b.

**Figure 6:**
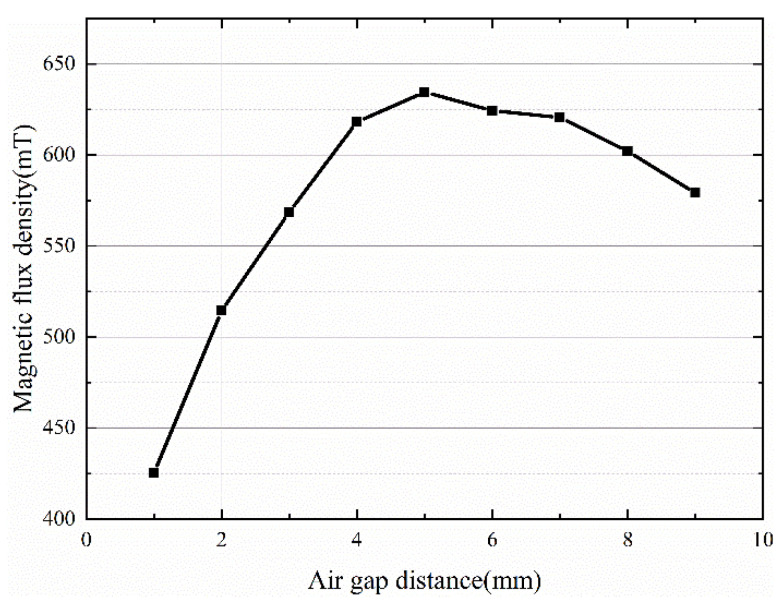
Influence of the air gap distance on the magnetic field.

Figure 6 shows the maximum magnetic flux density curve at the axis position when the chamfer angle is 45° and the air gap size is changed. When the air gap size is 1–5 mm, the magnetic flux density increases with increasing air gap size, and when the air gap size is 5 mm, the maximum magnetic flux density can reach 634.53 mT. When the air gap size is larger than 5 mm, the magnetic flux density decreases with increasing air gap size.

### 3.2. Effect of the Chamfer Angle

**Figure 7:**
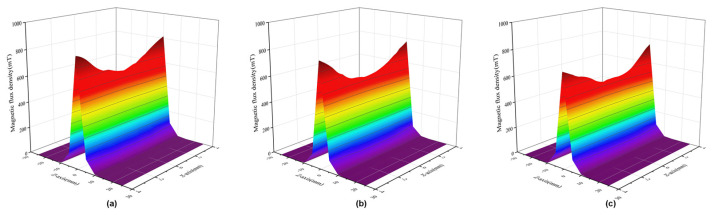
Influence of the chamfer on the magnetic field of the sensor: (**a**) 45° chamfer; (**b**) 60° chamfer; (**c**) 75° chamfer.

Figure 7 shows the magnetic flux density for the *z*-axis and the *x*-axis position when the air gap size is 5 mm and the angle of the chamfer is changed. Considering the structure of the sensor, this paper only analyzes situations for a chamfer of 45°, 60°, and 75°. It can be found that the maximum flux density in the *z*-axis direction is obtained when the angle is 45°, and the change in radial flux density is basically the same. In this paper, a sensor with a 5 mm air gap and 45° chamfer was selected, and it can be observed from the simulation results above that this choice helps the sensor achieve the highest flux density.

### 3.3. Effect of Relative Magnetic Permeability

**Figure 8:**
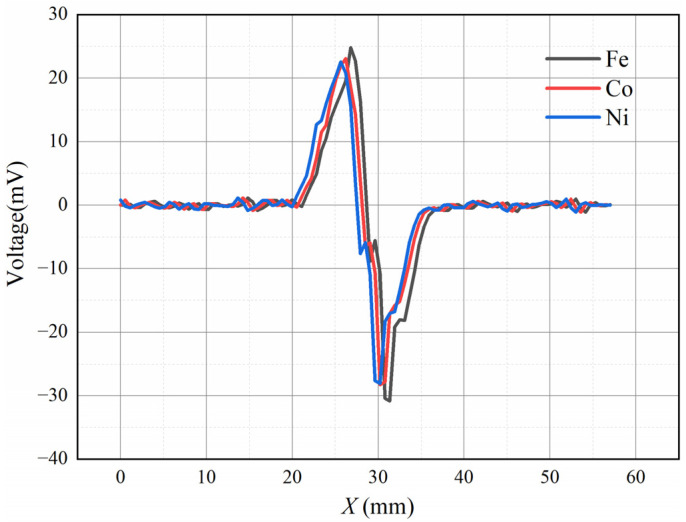
Influence of relative magnetic permeability.

The metals iron, cobalt, and nickel are relatively common ferromagnetic elements, and the properties of these three metals are shown in Table 2, where the relative permeability differs significantly. To investigate the effect of the relative permeability on the induced voltage signal, different kinds of square metal particles with a diameter of 0.5 mm were used to simulate the motion of wear debris in ANSYS Maxwell. The motion of wear debris in the radial direction of the flow channel was neglected, and the wear debris moved along the axial direction of the flow channel was at a speed of 1 m/s. The number of turns of the induction coil was set to 2000 turns with a single layer. The simulation results are shown in Figure 8. The waveform for the induced voltage is similar to a single-cycle sine waveform. The *V_pp_* values for the induced voltage are 55.64 mV (Fe), 51.36 mV (Co), and 50.67 mV (Ni), and the results show that the relative permeability of the ferromagnetic material at the same volume has little effect on the induced voltage signal.

### 3.4. Effect of Wear Debris Volume

**Figure 9:**
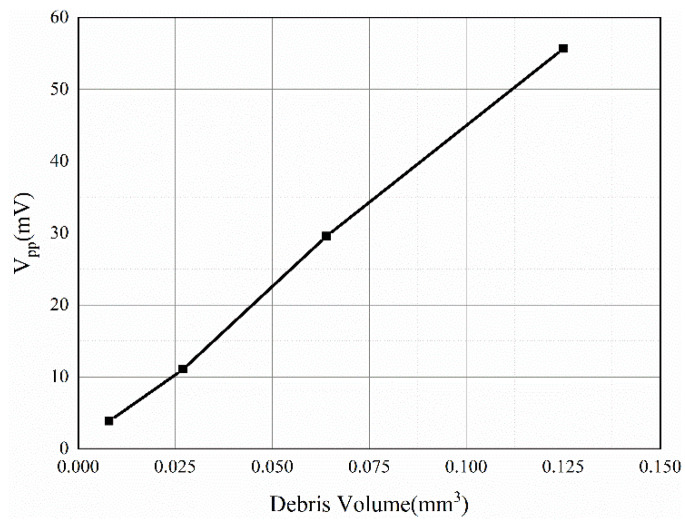
Influence of wear debris volume.

To investigate the influence of the debris size on the induction signal, square iron wear debris of different sizes (length of 0.2–0.5 mm) was used in ANSYS Maxwell, and it was assumed that the wear debris moves along the sensor axis at a uniform speed of 1 m/s. The number of turns of the induction coil was set to 2000 turns, and the number of layers was 1. The *V_pp_* value of the induced voltage signal of the wear debris at different sizes was obtained, and the *V_pp_* value of the signal was found to vary with the volume of the wear debris, as shown in Figure 9. It is obvious that the *V_pp_* value is approximately proportional to the volume of the wear debris, which is consistent with the mathematical model.

### 3.5. Effect of Wear Debris Velocity

**Figure 10:**
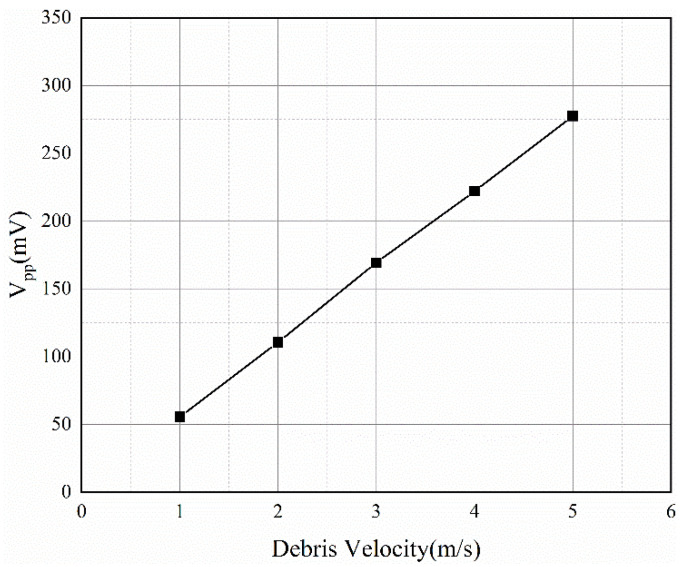
Influence of wear debris velocity.

In this simulation, the parameters other than the velocity parameters are guaranteed to be constant (square iron wear debris with a length of 0.5 mm, induction coil of 2000 turns, and 1 layer.) The simulation assumes that the wear debris moves in a uniform linear motion along the sensor axis, and speeds are set from 1 m/s to 5 m/s. Figure 10 shows the change in the *V_pp_* value with speed, and it can be concluded that the induced voltage for the wear debris is proportional to its speed of motion.

## 4. Experiment, Verification, and Discussion

**Figure 11:**
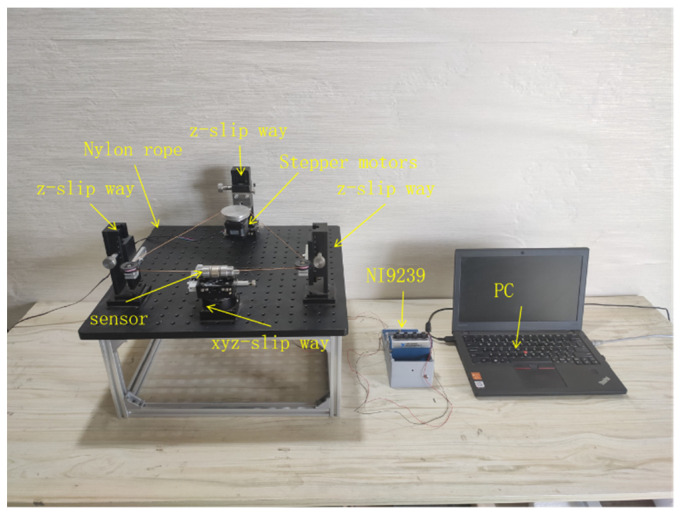
Single wear debris motion experimental rig.

The single wear debris experimental device is shown in Figure 11, which consists of a wear debris sensor, fixed pulley, stepping motor, and nylon rope. The experiment uses spherical iron particles as the wear debris, which are adhered to a nylon rope, and the stepper motor drives the nylon rope to make the wear debris move along the axis of the sensor flow channel. A slide table is placed under the sensor to adjust the position to ensure that the wear debris can move along the sensor axis position. In this paper, the induction coil is wound with 0.05 mm enameled wire, with 800 turns and a length of 8 mm (the induction coils adopted these parameters for all experiments unless stated otherwise). Since the induced voltage is relatively weak, the signal gain was adjusted by a voltage amplifier (amplification of 2000), and then an NI9239 instrument was used for voltage data acquisition.

### 4.1. Experimental Verification of Different Air Gap Distances

**Figure 12:**
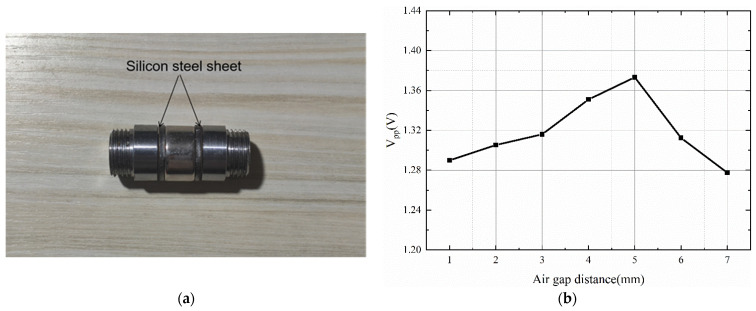
Experimental verification for different air gap: (**a**) silicon steel sheet adjusts the air gap distance of the sensor; (**b**) the *V_pp_* values of the induced voltage for different air gap.

For the sensor air gap verification experiment, the length of the air gap was adjusted by a silicon steel sheet, as shown in Figure 12a, and the wear debris was a spherical iron particle with a diameter of 0.33mm travelling at a speed of 1.48 m/s. The experimental results are shown in Figure 12b. The *V_pp_* value of the induced voltage of the wear debris is the largest when the sensor air gap is 5 mm, which means that the magnetic field strength of the oil tube is the largest when the air gap is 5 mm. This is consistent with the simulation results discussed above.

### 4.2. Experimental Verification for Different Relative Magnetic Permeabilities

**Figure 13:**
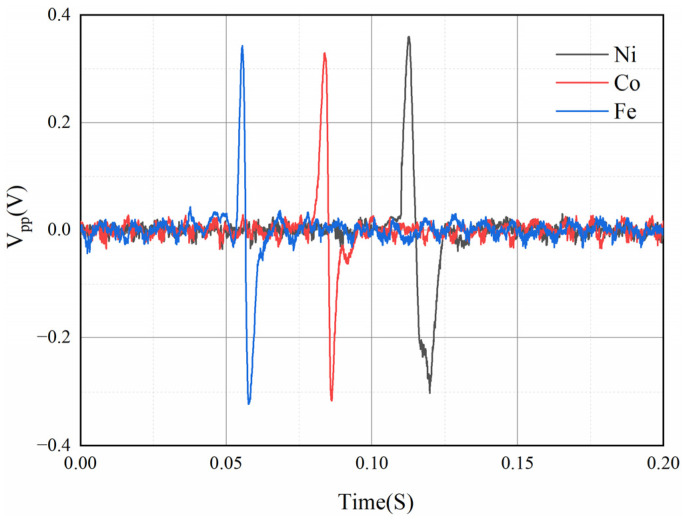
Induced voltage of three kinds of metals.

In this experiment, spherical iron, cobalt, and nickel metal particles with a diameter of 0.25 mm were selected, the parameters of the coil were kept constant, and the speed of the wear debris was 1.30 m/s. As shown in Figure 13, the induced voltages for the three metals does not differ much under the same size conditions. From Equation (7), the relative permeability of ferromagnetic wear debris was found to have a negligible effect on the induced voltage, which is consistent with the experimental results. Although this shows that the proposed sensor cannot distinguish the material of the ferromagnetic wear debris, the magnitude of the induced voltage generated for different ferromagnetic wear debris is not much different when other conditions are kept the same, so a quantitative analysis can be made for ferromagnetic wear debris.

### 4.3. Experimental Verification for Different Wear Debris Volumes

**Figure 14:**
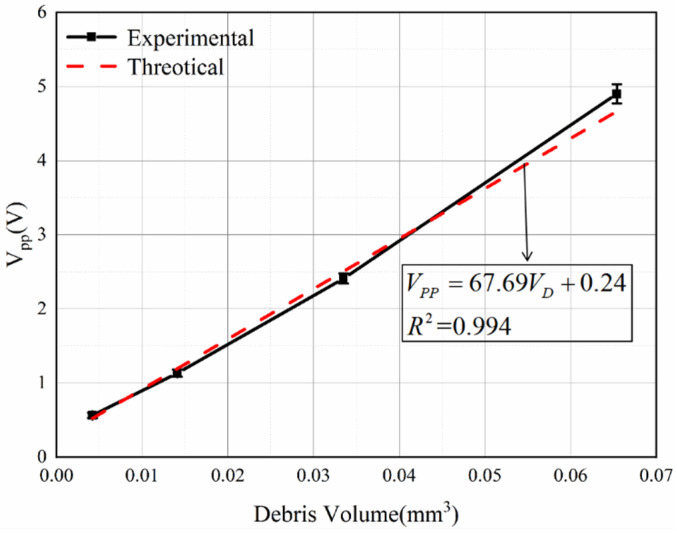
The *V_pp_* values of the induced voltage versus volume.

To investigate the effect of wear debris volume on the induced voltage signal, this paper used 0.2–0.5 mm spherical iron particles to conduct single wear debris experiments, in which the parameters for the coil and motor speed were kept constant and the size of the wear debris was changed. The experimental results are shown in Figure 14. At a speed of 1.48 m/s, the *V_pp_* value of the debris signal increases with increasing volume. Through a fitting analysis of the experimental data, it was found that there is an approximate linear relationship between the wear debris volume and the *V_pp_* value. There is a slight difference between the experimental data and theory. Because the induced signal in the experiment was easily affected by noise and voltage drift, the original signal data are directly counted in this paper without denoising.

### 4.4. Experimental Verification for Wear Debris with Different Velocties

**Figure 15:**
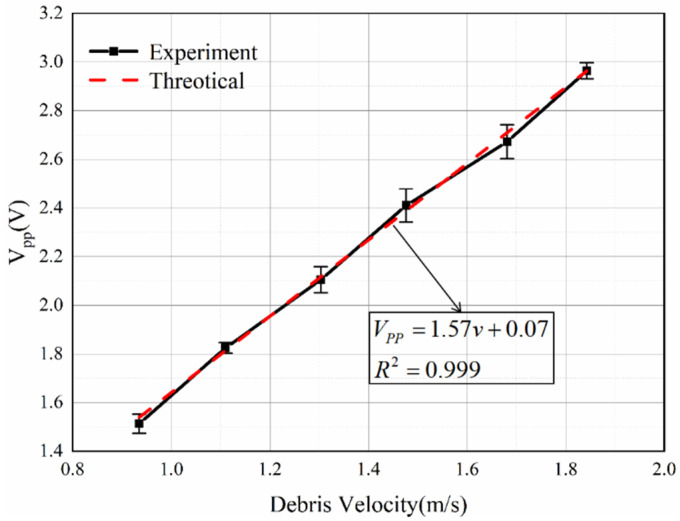
The *V_pp_* values of the induced voltage versus velocity.

In this experiment, the coil parameters were guaranteed to be constant, and the wear debris diameter was 0.4 mm. The speed of the wear debris was controlled by a stepper motor. Six sets of speeds were set: 0.93 m/s, 1.11 m/s, 1.30 m/s, 1.48 m/s, 1.68 m/s, and 1.84 m/s. Figure 15 shows that there is a linear relationship between the wear particle velocity and the value for the *V_pp_* signal. The difference between the experimental results and theoretical results is mainly because the induced voltage signal in the experiment is affected by noise and voltage drift.

### 4.5. Comparation with Other Electromagnetic Sensors

This paper proposes a permanent magnet axisymmetric high-gradient magnetic field inductive wear debris sensor with a compact sensor structure, no external power supply, no energy loss, and a stable magnetic field generated by permanent magnets. Moreover, it has a higher detection accuracy for ferromagnetic wear particles, to solve the problem of magnetic field instability and excessive volume of inductive sensors for other types of electromagnetic sensors. The comparation with other electromagnetic sensors is listed in Table 3.

## 5. Sensor Calibration

As shown in Figure 14 and Figure 15, the volume and velocity of the wear debris are not strictly proportional to the induced voltage. There are three main reasons for this: the first is the existence of errors in the magnetization of the permanent magnets as well as assembly errors, the second is that the induced signal is affected by electromagnetic noise and voltage drift, and the third is the error introduced by the amplifier. Therefore, it is necessary to modify the theoretical value according to the experimental value and complete the calibration of the sensor.

From Equation (7), the inducted voltage is found to be related to the volume and velocity of the wear debris. Experiments show that the *V_pp_* value of the induced voltage is proportional to the volume or velocity of the wear debris. Therefore, the *V_pp_* value for the induced voltage can be expressed as:

(8) $$V_{pp}=kV_{D}v$$

where k is a constant to be measured, $\;V_{D}$ is the volume of the wear debris.

**Figure 16:**
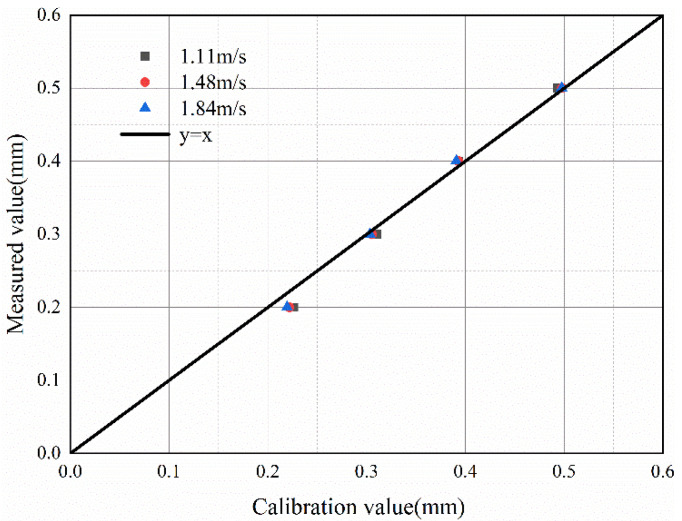
Comparison of experimental value and theoretical value.

The experimental data are shown in Table 4. The amplifier gain was 2000. To exclude noise interference, the value of the induced voltage u should be taken as the difference between the value of *V_pp_* and the noise. From substituting the experimental data into Equation (8), k=0.0257±0.0036. As shown in Figure 16, the theoretical value obtained from the sensor calibration is not much different from the experimental value, which proves the correctness of the sensor calibration.

## 6. Detection Accuracy of Ferromagnetic Particle

**Figure 17:**
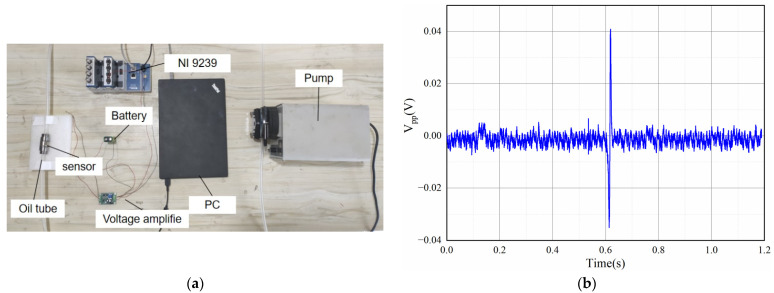
Lube oil experiment: (**a**) wear debris monitoring test bench; (**b**) results obtained from the oil experiment.

To verify the actual detection effect of the sensor, a wear debris monitoring experiment bench was established, as shown in Figure 17a, which consists of a peristaltic pump, a NI 9239 acquisition card, an amplifier (2000 times), a laptop computer, and a sensor. The coil has 1200 turns, 8 mm length, and 3 layers. The oil speed was adjusted by a peristaltic pump, and the wear debris flows through the sensor with the oil to generate the induced voltage. The signal detected by the sensor for 50 μm iron wear debris travelling at an oil speed of 500 mL/min is shown in Figure 17b; the induced voltage signal is obvious, and the induced voltage shows a *V_pp_* value of 0.076 V.

## 7. Conclusions

In this paper, a permanent magnet axisymmetric high-gradient magnetic field wear debris sensor is proposed. Compared with other types of electromagnetic sensors, this sensor has a smaller size, is prone to less interference, and has a higher detection accuracy for ferromagnetic wear particles. A mathematical model of the sensor is proposed, and the influence of various parameters on the sensing performance is analyzed by software simulation. The correctness and rationality of the model and simulation results were verified in a single wear debris experiment. The optimal air gap was selected, and it was concluded that the induced voltage is little affected by the relative permeability of the wear debris and is proportional to the volume and velocity of the wear debris. Then, the calibration of the sensor was completed according to the experimental data, and a quantitative analysis of the wear debris was realized. Finally, the detection performance of the sensor was tested using a lube oil wear debris monitoring test bench, where the sensor was able to detect 50 μm ferrous wear debris in lube oil at a flow rate of 500 mL/min.

## Figures and Tables

**Table 1:** Parameters discussed in the simulation.

Parameter	Explanation	Value
M	Material of permanent magnet	NdFeB
$D_{g}$	Air gap distance	1, 2, 3, 4, 5, 6, 7, 8, 9 mm
$A_{c}$	Chamfer angle	45°, 60°, 75°
$\mu_{r}$	Relative Magnetic Permeability	Fe (7000), Co (174), Ni (1120)
$S_{d}$	Square iron wear debris of different sizes	length of 0.2–0.5 mm
$v_{d}$	Wear debris velocity	1, 2, 3, 4, 5 m/s

**Table 2:** Properties of wear debris.

Metal Material	Density (g/cm^3^)	Relative Magnetic Permeability
Fe	7.86	7000
Co	8.9	174
Ni	8.9	1120

**Table 3:** Comparation with other sensors.

Ref	Minimum Detectable Ferro-Particle Size (μm)	Diameter of Oil Tube (mm)	Easy to Fabricate	Easy to Extract Signal
[32]	100	7.6	NO	NO
[33]	16	0.5	NO	NO
[30]	30	2	YES	YES
This paper	50	6	YES	YES

**Table 4:** Experimental data.

V_D_ (mm^3^)	v (m/s)	*V_pp_* (V)
	1.1102	0.4476 ± 0.0324
0.0042	1.4761	0.5630 ± 0.0358
	1.8427	0.6840 ± 0.0294
	1.1102	0.8945 ± 0.0341
0.0141	1.4761	1.1334 ± 0.0492
	1.8427	1.3848 ± 0.0479
	1.1102	1.8247 ± 0.0217
0.0335	1.4761	2.4099 ± 0.0680
	1.8427	2.9636 ± 0.1031
	1.1102	3.5967 ± 0.0575
0.0654	1.4761	4.8997 ± 0.1299
	1.8427	6.1112 ± 0.3113

## Data Availability

Not applicable.
